# Blobology: exploring raw genome data for contaminants, symbionts and parasites using taxon-annotated GC-coverage plots

**DOI:** 10.3389/fgene.2013.00237

**Published:** 2013-11-29

**Authors:** Sujai Kumar, Martin Jones, Georgios Koutsovoulos, Michael Clarke, Mark Blaxter

**Affiliations:** ^1^Institute of Evolutionary Biology, Ashworth Laboratories, University of EdinburghEdinburgh, UK; ^2^Edinburgh Genomics, University of EdinburghEdinburgh, UK

**Keywords:** next-generation sequencing, metagenomics, assembly, parasites, symbionts, commensals, contaminants

## Abstract

Generating the raw data for a *de novo* genome assembly project for a target eukaryotic species is relatively easy. This democratization of access to large-scale data has allowed many research teams to plan to assemble the genomes of non-model organisms. These new genome targets are very different from the traditional, inbred, laboratory-reared model organisms. They are often small, and cannot be isolated free of their environment – whether ingested food, the surrounding host organism of parasites, or commensal and symbiotic organisms attached to or within the individuals sampled. Preparation of pure DNA originating from a single species can be technically impossible, but assembly of mixed-organism DNA can be difficult, as most genome assemblers perform poorly when faced with multiple genomes in different stoichiometries. This class of problem is common in metagenomic datasets that deliberately try to capture all the genomes present in an environment, but replicon assembly is not often the goal of such programs. Here we present an approach to extracting, from mixed DNA sequence data, subsets that correspond to single species’ genomes and thus improving genome assembly. We use both numerical (proportion of GC bases and read coverage) and biological (best-matching sequence in annotated databases) indicators to aid partitioning of draft assembly contigs, and the reads that contribute to those contigs, into distinct bins that can then be subjected to rigorous, optimized assembly, through the use of taxon-annotated GC-coverage plots (TAGC plots). We also present Blobsplorer, a tool that aids exploration and selection of subsets from TAGC-annotated data. Partitioning the data in this way can rescue poorly assembled genomes, and reveal unexpected symbionts and commensals in eukaryotic genome projects. The TAGC plot pipeline script is available from https://github.com/blaxterlab/blobology, and the Blobsplorer tool from https://github.com/mojones/Blobsplorer.

## INTRODUCTION

The raw power of new sequencing methods has permitted the expansion of genome science into a wide range of new biological systems. In particular the technologies permit genome sampling from wild organisms and communities of organisms. This approach was unthinkable in the era of Sanger-sequenced genomes, as the per-base cost precluded deep sampling of mixed starting materials in order to assemble the genome or transcriptome of a particular target organism. However most species of interest are not easily separable from their environments, either because they cannot yet be cultured cleanly, or because they are very intimately involved with a host or other commensal and parasitic organisms.

In our research program, focused on the genome biology of the phylum Nematoda and related animals (Blaxter et al., 2012; Godel et al., 2012; Kumar et al., 2012; Wang et al., 2012), we are frequently faced with DNA samples and thus genome sequence datasets from wild isolates of target species where a significant proportion of the sequence data derives from the non-nematode components of the ecosystem. For example, tissue-dwelling nematodes often ingest the cells of their host animals or plants, and immune reactions can involve the adherence and crosslinking of host cells to parasite surfaces. Even free-living nematodes, feeding on bacteria or fungi, can come with attached or ingested food, as difficult-to-remove biofilms, or sequestered in the animals’ intestines. These mixed samples are akin to low-complexity metagenomes, where a metagenome samples all the replicons present in an ecological sample. We have frequently observed DNA samples that are “contaminated” with the genomes of other species: components of food, commensal organisms, parasites and pathogens, or laboratory contaminants. It is particularly common to observe bacterial genomic contamination of eukaryotic samples.

The research goals of these projects require the removal (or at least positive identification) of the data that derive from non-target genomes. Inadvertent inclusion of reads from accidentally or unavoidably sampled bacterial or parasite data in a genome assembly could result in target genome mis-assembly, reductions in the overall quality of the genome assembly, or even attribution of the non-target genetic material (and the genes and functions inferred from the sequence) to the reported target genome. There are several issues that preclude simple co-assembly of raw low complexity metagenome data. The first is that most assemblers, and particularly de Bruijn assemblers, assume a particular modal read coverage of the genome to be assembled. If the contaminating genomes are at different molar concentrations then the internal logic of the assembler may optimize the output to an erroneous modal coverage. For example, a raw read dataset of a parasite of vertebrates might contain 45% parasite, 45% host, and 10% bacterial reads. If the parasite genome is 100 Mb, the vertebrate 3000 Mb and the bacterium 5 Mb, the genomes will be present at molar ratios of one parasite to 0.03 host to approximately five bacterium. Assemblers will find the bacterial replicons easier to assemble, at the expense of the desired parasite genome. Secondly, different genomes can have very different inherent “assembleability,” and in particular bacterial genomes (with high proportional content of protein-coding sequence, and low repeat content) are more easily assembled than are highly repetitive and gene-poor eukaryotes. Lastly, different genomes can have very different proportions of G and C bases, and mixing low GC genome data with balanced GC genome data may result in assemblies biased toward the mid-GC range.

We here present an effective solution to these problems. By performing a very preliminary assembly, with no attempt to optimize the output, and then classifying the resulting contigs by coverage (a proxy for relative molarity of the genomes in the mix), relative GC content (separating genomes with distinct biases), and best similarity match in public databases (separating data by likely species of origin), we can divide the raw data into bins that can be optimally assembled independently. We have used these methods to clean up “contaminated” DNA samples, identify data of interest in difficult-to-disentangle host–parasite systems, and extract intracellular bacterial symbiont genomes from within a whole-organism dataset (Kumar and Blaxter, 2011; Godel et al., 2012). This idea is not unique to our group’s work, and has been proposed previously for cleaning of Roche 454 sequence datasets from microbial communities (Nederbragt et al., 2010) and assembly of individual genomes from bacterial associates of plants (D’haeseleer et al., 2013). Here we present an improved version of our pipeline for exploration of taxon-annotated, GC-coverage plots (TAGC plots; Kumar and Blaxter, 2011; Godel et al., 2012) and a graphical tool for TAGC plot exploration, Blobsplorer. The TAGC plot/Blobsplorer toolkit is coded in Perl, R and JavaScript, and includes a graphical interface for exploring the distributions of read coverage, GC, and sequence similarity in large next-generation datasets.

## MATERIALS AND METHODS

### EXAMPLE DATA: SEQUENCING

*Caenorhabditis* sp. 5 (strain JU800) DNA was provided by Asher Cutter (University of Toronto). The DNA was extracted from a sucrose- and detergent-cleaned plate culture of nematodes using proteinase K and phenol–chloroform. The standard Illumina protocol was used to generate two libraries with fragment sizes 300 and 600 bp and sequenced on an Illumina HiSeq2000 instrument using 101 base, paired-end sequencing with V3 reagents. Raw sequence data are available at the Short Read Archive with accession number ERP001495. Raw reads were adapter- and quality-trimmed using fastq-mcf (Aronesty, 2011; **Table 1**) with a trimming threshold quality of 20, discarding reads shorter than 50 b. A total of 136.3 M read pairs totaling 26.9 Gb remained after these trimming steps (**Table 2**). A full analysis of the genome of *Caenorhabditis* sp. 5 will be published elsewhere. The *Dirofilaria immitis* sequencing data have been described previously (Godel et al., 2012).

**Table 1:** Software and databases used in this work.

**Tool or resource name**	**Version**	**Reference**	**Source website**	**Additional parameters used**	**Comments**
**Data QC/filtering**					
fastq-mcf	1.04.636	Aronesty (2011)	http://code.google.com/p/ea-utils/wiki/FastqMcf		
**Preliminary assembly and read mapping**					
ABySS	1.3.6	Simpson etal. (2009)	http://www.bcgsc.ca/platform/bioinfo/software/abyss	k-mer of 61	The user might care to change the k-mer value depending on the quality and length of their read data; it is not necessary to optimize this value. The program can also be run treating any paired (mate or paired-end) data as single-end.
Bowtie 2	2.1.0	Langmead and Salzberg (2012)	http://bowtie-bio.sourceforge.net/bowtie2/index.shtml	-k 1 –very-fast-local	The settings used are designed to map reads uniquely and quickly
**Taxonomic annotation**					
BLAST+	2.2.28	Ye et al., 2006; Johnson et al., 2008	http://blast.ncbi.nlm.nih.gov/Blast.cgi?CMD=Web&PAGE_TYPE=BlastDocs&DOC_TYPE=Download	-task megablast -evalue 1e-5 -max_target_seqs 1 -outfmt ‘6 qseqid staxids’	
NCBI nt	March 1, 2013		ftp://ftp.ncbi.nlm.nih.gov/blast/db/		See http://blast.ncbi.nlm.nih.gov/Blast.cgi?CMD=Web&PAGE_TYPE=BlastDocs&DOC_TYPE=ProgSelectionGuide for definition; one can also use custom databases, or other normalized databases
**TAGC plot scripts**					
gc_cov_annotate.pl	1.0	This work	https://github.com/blaxterlab/blobology		
makeblobplot.R	1.0	This work	https://github.com/blaxterlab/blobology	0.01 taxlevel_order	0.01 is the threshold of displaying annotated contigs, and taxlevel_order sets the taxon level to display
ggplot2		Wickham (2009)	http://ggplot2.org/		
NCBI taxonomy heirarchy files	March 2013		ftp://ftp.ncbi.nlm.nih.gov/pub/taxonomy/taxdump.tar.gz		
**Blobsplorer**					
JQuery	1.8.2	http://jquery.com/	http://code.jquery.com/jquery-1.8.2.js		Additional JQuery plugins used: jquery-ui, dropkick, tagsinput, placeholder, chardin.js
Raphael	2.1.0	http://raphaeljs.com/	http://github.com/DmitryBaranovskiy/raphael/raw/master/raphael-min.js		additional Raphael plugins used: raphael.export
**Assembly validation**					
*Caenorhabditis briggsae* proteome	WS230	Stein etal. (2003)	ftp://ftp.wormbase.org/pub/wormbase/species/c_briggsae/sequence/protein/		See http://www.wormbase.org/species/c_briggsae#02–10
*Caenorhabditis* sp. 5 EST assembly	NEMBASE4	Elsworth etal. (2011)	http://www.nematodes.org/downloads/databases/NEMBASE4/CSC_nuc.fsa		See http://www.nematodes.org/nembase4/species_info.php?species=CSC
*Caenorhabditis* sp. 5 RNA-Seq transcriptome assembly	1.0		http://nematodes.org/genomes/caenorhabditis_sp5/index.html		Unpublished data from the *Caenorhabditis* sp. 5 genome project
CEGMA	2.4	Parra etal. (2007)	http://korflab.ucdavis.edu/datasets/cegma/		

**Table 2:** Sequence data for *Caenorhabditis* sp. 5.

**Nematode strain identifier**	**Library insert size (bp)**	**Type of sequencing**	**Number of raw reads**	Number of bases in raw reads (Gb*)	**Number of reads after trimming**	**Number of bases in trimmed reads (Gb)**	**ERA accession**
JU800	300	HiSeq2000 101 b PE^*^	88.6 M^*^ pairs	17.9	86.9 M pairs	17.3	ERR138445
JU800	600	HiSeq2000 101 b PE	52.4 M pairs	10.6	49.4 M pairs	9.6	ERR138446

* Gb, gigabases; PE, paired end; M, million.

### TOOLS USED IN THE TAGC PLOT PIPELINE

The TAGC plot pipeline uses a number of external tools (**Table 1**). Some of the external tools are easily substituted with the user’s preferred option. The core processing is carried out using a Perl script, *gc_cov_annotate.pl* and an R script *makeblobplot.R* (**Table 1**). The output includes a tab-separated value (TSV) format file with a single header row followed by one row per contig. The first three columns of each row give the sequence ID, length, and GC content. There follow an arbitrary number of columns, whose field headers begin with the string “cov_,” giving the coverage for each library. After these come an arbitrary number of taxonomic annotation columns, whose field headers begin with the string “taxlevel_.”

### BLOBSPLORER

Blobsplorer takes as input the text file produced by *gc_cov_annotate.pl*. The tool can process and display text files from any source as long as they conform to the format defined above. Blobsplorer is implemented as a single web page, with the processing and visualization code written in JavaScript. JQuery is used to update the plot in response to interface events and Raphael to draw the plot itself. Blobsplorer uses the HTML5 file API, allowing it to be distributed as a static web page which does not require a server-side component: all processing is carried out by the browser, so the tool can be run simply by opening a local copy of the page.

## RESULTS

### OVERVIEW OF THE TAGC PLOT (OR BLOBPLOT) METHOD

**FIGURE 1:**
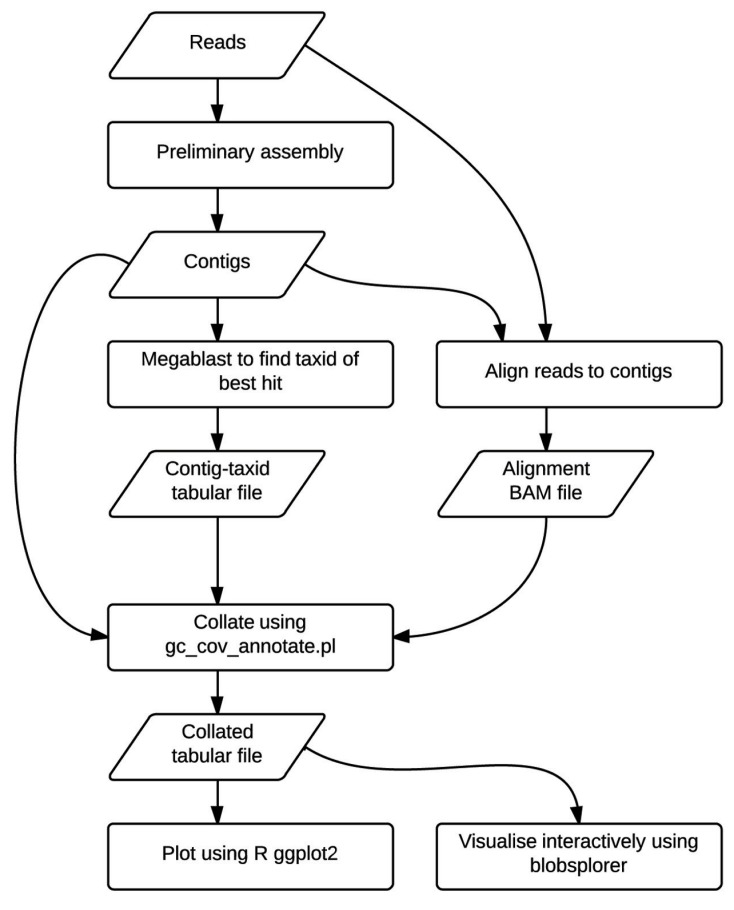
**The TAGC plot workflow.** Flowchart showing analysis steps and intermediate data files in the TAGC plot workflow. Rectangles indicate processing stages, while parallelograms represent data files.

The TAGC plot method is simple to perform (**Figure 1**). The user first collects and filters their raw genome sequencing data as for any standard assembly project. A preliminary assembly is then generated, without any attempt to optimize parameters. This assembly serves to reduce the complexity of the data from tens or hundreds of millions of short reads down to tens or hundreds of thousands of longer, contiguated sequences (contigs). The reduced complexity dataset is easier to screen, partly because of the smaller number of analytic steps needed, but also because the longer sequences are a better substrate for assessment of numerical (GC proportion, coverage) and biological (similarity to known sequences) metrics. The method is agnostic as to which assembler is used for this step. In this paper we present use of ABySS (Simpson et al., 2009), but we have also used Velvet (Zerbino and Birney, 2008) and CLCBio assemblyCell (see http://www.clcbio.com/products/clc-assembly-cell/) in the past. There is no need to extensively scaffold the assembly, and we have used mate-pair data given to the assembler as “single-end” for TAGC plot analyses in the *D. immitis* example.

The average GC content of each contig in the preliminary assembly is calculated. The raw reads are mapped back to this preliminary assembly and the resulting alignment BAM file used to calculate average read-depth coverage for each contig. We use Bowtie 2 (Langmead and Salzberg, 2012) here. It is also possible to use other read mappers that output BAM format, or to use read or k-mer coverage metrics reported by the assembler directly. The contigs from the preliminary assembly are compared to the NCBI non-redundant nucleotide (nt) database using the megablast option in the BLAST+ suite (Ye et al., 2006; Johnson et al., 2008) to identify a best species hit. It is also possible to construct custom local databases if the taxonomy of the “contaminants” is known, but use of the complete NCBI nt database is recommended as this also results in detection of unexpected contaminants. GC content, read-coverage, and taxonomic information are then combined to generate a standard format file, which is visualized as a TAGC plot. The TAGC plot is then reviewed, and strategies for removal of contaminants, extraction of required reads, and other binning operations defined. The TAGC plot data can also be viewed in Blobsplorer, a JavaScript tool that permits exploration and selection of contig sets interactively in a web browser.

### EXAMPLE OF TAGC PLOT USE IN FILTERING DATA FOR ASSEMBLING *Caenorhabditis* sp. 5

Here we demonstrate the use of the method to generate TAGC plots for the sequencing of the free-living nematode *Caenorhabditis* sp. 5 (see http://nematodes.org/genomes/caenorhabditis_sp5/). *Caenorhabditis* sp. 5 is an as-yet unnamed species, found in eastern Asia, a member of the *briggsae* subgroup of the genus *Caenorhabditis* (Kiontke et al., 2011). All the scripts used are available at https://github.com/blaxterlab/blobology along with an accompanying bash script that can be run to replicate the results below, or modified to run the pipeline on a different read set.

A preliminary assembly was performed on the adapter- and quality-trimmed reads using ABySS (Simpson et al., 2009) with default options and a k-mer of 61 with the 300 and 600 bp libraries provided as separate inputs. We used ABySS because it is open-source, modular, and highly parallelizable. One of the advantages of ABySS is that it does not require the user to provide an *a priori* fragment-size estimate as the tool works out the fragment sizes for each library based on its own mapping of reads to an initial unitig assembly. Empirical verification of library insert sizes is a useful, additional quality-control step. We did not attempt to optimize k-mer, coverage cutoff, or other assembly parameters, as the goal of the preliminary assembly is only to reduce the scale of the dataset for taxonomic identification and to estimate coverage. As ABySS uses read pair data in assembly, the output assembly file sequences will include unresolved bases (“N”) that link contigs spanned by read pairs. Thus the contigs we assess might strictly be considered “scaffolds.” The final ABySS assembly FASTA format file was filtered to remove sequences smaller than 200 bp, resulting in 12,264 contigs with an N50 (length-weighted median) of 32,806 bp and a mean length of 13,125 bp, spanning 161.0 Mb. The expected size of the *Caenorhabditis* sp. 5 genome is ~130 Mb.

We note that different assemblers have inbuilt low-coverage cutoff parameters. For example, ABySS, used here, has a filter to discard contigs with a k-mer coverage less than the square root of median coverage, while CLCBio assemblyCell has a coverage cutoff of 2. Thus different assemblers may return very different numbers of contigs from the same input data solely due to their handling of low coverage contigs. While these contigs will tend to be shorter than higher-coverage contigs, they can contribute significantly to assembly span, and depress the N50 and (especially) the mean lengths of assemblies. These “extended” assemblies may score better on some biological measures of completeness, but our experience is that, given sufficient (i.e., >60-fold) coverage of the target genome, discarding these short, poorly supported contigs is advantageous.

Read coverage can be derived directly from the assembler output (for example both ABySS and Velvet report coverage metrics in the FASTA headers of the output files). We wanted to review the coverage and contamination statistics for each of our libraries separately, to permit detection of per-sample or per-library contaminants, and so remapped all data using Bowtie 2. Any read alignment tool that produces a BAM file can be used, as the downstream tools simply need an assembly FASTA file and an alignment BAM file. Mapping was performed using the settings -k 1 (max number of matches per query) and -*-very-fast-local* because the goal was to get an estimate of read coverage rapidly, and not to get the most precise or sensitive mapping. For the *Caenorhabditis* sp. 5 data, 98.69% of all reads mapped back to the preliminary assembly.

### TAXONOMIC ANNOTATION OF THE PRIMARY ASSEMBLY

We identified the taxonomic attribution of the best-matching sequence in the NCBI nt database using BLAST+ *megablast* (Ye et al., 2006; Johnson et al., 2008). We generated a two-column table with the contig ID in the first column and the taxonomy ID of the species of origin of the best hit (lowest *e*-value) using the BLAST+ output formatting controls (see **Table 1**). Other tools such as MEGAN (Huson et al., 2007; Huson and Weber, 2013) or exonerate (Slater and Birney, 2005) might have provided more accurate results, but BLAST+ is convenient because it is very fast, natively parallel, and provides species taxonomy IDs in tabular form in one step. While we queried all 12,264 sequences in the preliminary *Caenorhabditis* sp. 5 assembly against NCBI nt, a randomly selected subset from preliminary assemblies with many hundreds of thousands of assembled sequences can speed up this part of the process with little reduction in final ability to screen for contaminants.

### MAKING AND INTERPRETING TAGC PLOTS

**FIGURE 2:**
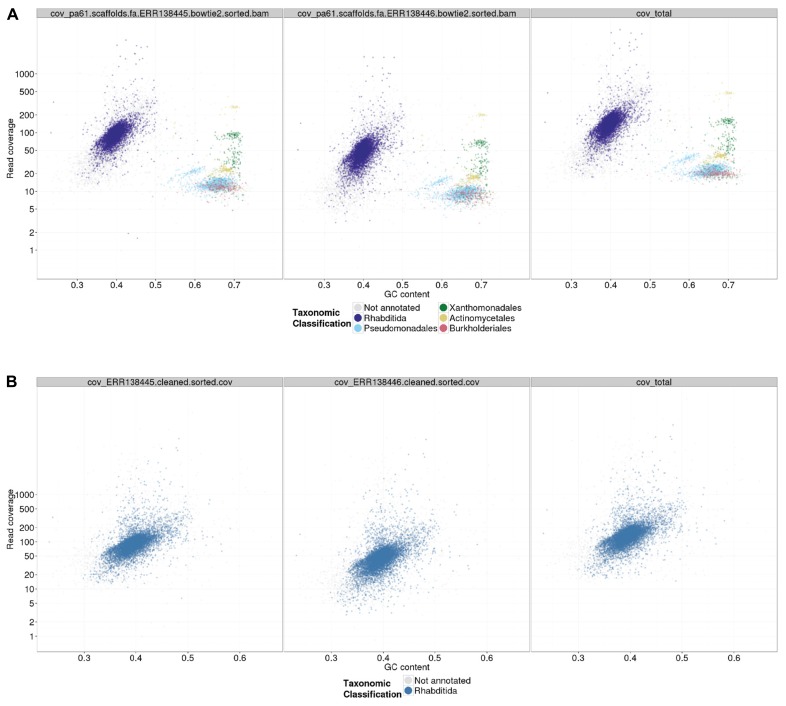
**TAGC plot of *Caenorhabditis* sp. 5 preliminary assembly.** **(A)** A TAGC plot was constructed as described in the text from the ABySS assembly of the full Illumina dataset for *Caenorhabditis* sp. 5. The three panels are (left) 300 bp library, (middle) 600 bp library, and (right) both libraries combined, mapped to an assembly that used the combined data. Individual contigs are plotted based on their GC content (*x*-axis) and their read coverage (*y*-axis; logarithmic scale). Contigs are colored according to taxonomic order of their best megablast match to the NCBI nt database (with *E*-value cutoff < 1e-05). Any taxonomic order annotation associated with 1% or more of annotated contigs is marked with a color; contigs without an annotation from these are in gray. **(B)** The TAGC plot from an ABySS assembly of the *Caenorhabditis* sp. 5 data after removal of the bacterial contaminants. Annotation as in part **(A)**.

**FIGURE 3:**
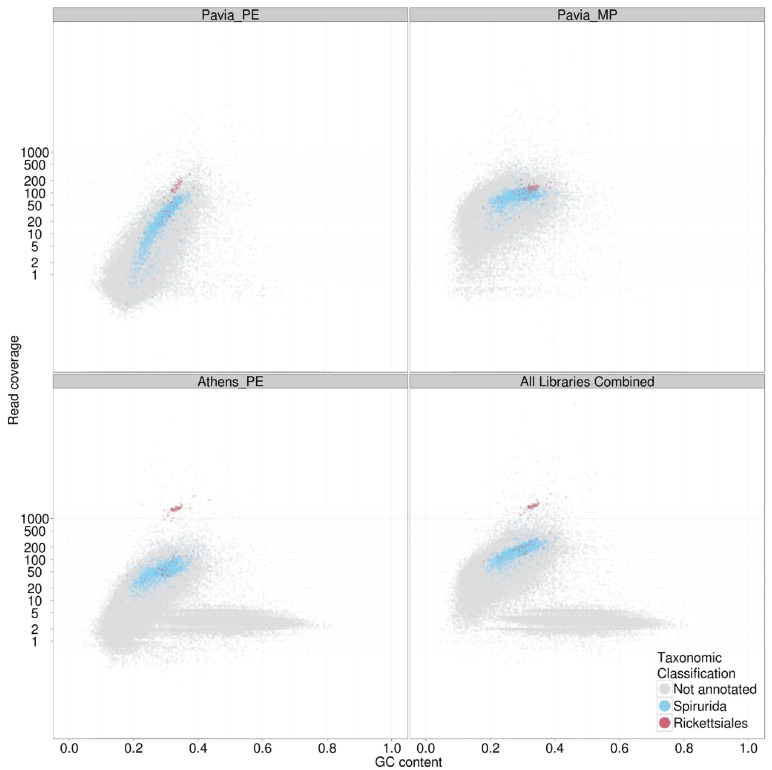
**TAGC plot of *Dirofilaria immitis* and its *Wolbachia* endosymbiont.** The four panels display TAGC plots for (upper left) a paired end library form the “Pavia” male nematode, (upper right) a mate pair library from the “Pavia” male nematode, (lower left) a paired end library from the “Athens” female nematode and (lower right) all data combined. The specified read sets were aligned to an assembly generated from the paired end data. The TAGC plots were taxonomically annotated, and contigs with best similarity to Spirurida (the order to which *D. immitis* belongs) and Rickettsiales (the alphaproteobacterial order to which *Wolbachia* belongs) were highlighted in color. Other conventions as in **Figure 2**.

A custom Perl script, *gc_cov_annotate.pl*, was used to collate the three input types: the assembly FASTA file, the alignment BAM files, and the tabular sequence-to-species mapping file, and produce a single data file that was visualized using the ggplot2 graphics library (Wickham, 2009) in R. The output (**Figures 2** and **3**) includes separate panes for each library read file and colors contigs plotted in the GC-coverage space by the most abundantly represented taxa matched. Unmatched contigs are shaded gray. In the case of the *Caenorhabditis* sp. 5 TAGC plots (**Figure 2A**), there were no major differences between the two independent libraries other than in average read depth, as expected. The TAGC plots show a major “blob” of contigs with high (~100-fold) coverage and 35–55% GC, with predominant taxonomic identification as Rhabditida (the order containing *Caenorhabditis*). The apparent skew in this blob, with contigs of lower mean GC having lower coverage, is typical of Illumina datasets, as there are biases due to library preparation and solid-phase PCR that result in under-representation of low GC sequences. Note also that there are some contigs, annotated as Rhabditida, with very high coverages (up to 2000-fold). These represent either repeats, or the mitochondrial genome. To the right, at higher GC, are a set of blobs with distinct coverage means, and distinct consistent taxonomic assignments (to orders of bacteria, including Pseudomonadales, Xanthomonadales, Actinomycetales, and Burkholderiales). These blobs derive from contaminating bacterial species, some at low levels (Pseudomonadales at ~10-fold, or one genome to every 10 *Caenorhabditis* sp. 5 genomes) and some at higher levels (such as Actinomycetales at ~200-fold coverage). To aid visualization, if the number of contigs assigned a specific taxonomic identification is less than 1% of the total number of annotated contigs, that taxon is not shown in the legend.

### BLOBSPLORER: INTERACTIVE TAGC PLOTS

To aid exploration of TAGC plots, we have developed an interactive tool, Blobsplorer, for investigation of TAGC plots. Blobsplorer is written in JavaScript and consists of a single static web page. All processing is carried out client-side and requires no additional dependencies. Because next-generation assemblies tend to have large numbers of contigs (particularly for mixed-species data), a degree of sampling is usually necessary to allow the interface to update in a responsive manner. In testing, we found that a desktop computer can comfortably display data for around 20,000 contigs. Blobsplorer has the ability to sample data points when loading input data files.

The Blobsplorer workflow is straightforward. After selecting an input file, the user chooses a sampling level from the drop-down menu before clicking “load contigs.” A point is then plotted on the TAGC plot for each sampled contig. The taxonomic level at which coloring is applied can by changed by selecting a value from the “color by” drop-down menu. Clicking the “Download as SVG” button will generate a copy of the plot in scalar vector graphics (SVG) format, which can be opened in a scalar vector drawing package for further processing (for example, to create publication-ready graphics). Once the data have been loaded and displayed, groups of contigs can be defined by drawing ellipses on the plot. To draw an ellipse, the user clicks once on the plot to define the center, and then moves the cursor to define the shape of the ellipse. They then click a second time, and move the cursor to define the rotation of the ellipse. Clicking for the third and final time on the plot completes the definition of the ellipse. Multiple ellipses can be drawn in this way to define a set of contigs. Clicking the “highlight selected” button will confirm the selection visually by shading the selected contigs in red, while clicking the “download contig ids” button will generate a text file containing the identifiers of the selected contigs which can be downloaded for further processing. Clicking the name of a taxonomic group in the taxonomic annotation key will cause points assigned to that group to be highlighted.

### BINNING THE RAW DATA: POSITIVE AND NEGATIVE FILTERS

Following TAGC plot visualization, our approach is to devise a contig and read selection strategy that will bin data for the desired target organism(s) separately from any contaminants. This separation then permits exploration and optimization of assembly parameters focused on the raw data coverage, and biological idiosyncrasies, of one genome at a time. We devised a mix of positive and negative filters to keep contigs that were likely to derive from the nematode genome (because of their taxonomic annotation, coverage, or GC) and to exclude contigs (using the same criteria but focusing on the bacterial blobs). In the case of *Caenorhabditis* sp. 5, we used a simplified database of proteobacterial sequences (all the contaminants were proteobacteria) to identify contigs deriving from the contaminants. In addition we identified contigs with GC and coverage similar to the identified proteobacterial contaminants. To produce a cleaned read set for high-quality assembly, we identified and removed the reads that mapped to contaminant contigs, and then also identified the pair reads of any unpaired reads in this set, and collected the reads (and pairs) that mapped to these. This set of reads was removed from the filtered raw data before reassembly. Scripts and commands for selecting contigs by various criteria, and the reads that map to them, are provided in the GitHub repository. The reassembly was again screened using TAGC plots to confirm that the read cleaning process had been effective (**Figure 2B**). We have found that second-round assemblies can sometimes reveal novel or further contaminant contigs. This is likely to be due to sequence data that failed to be assembled by the de Bruijn assembler in the first round because of conflicts or interference between different possible paths in the assembly graph. In many cases these second-round-identified contaminants have only been visible as blobs of distinct GC and coverage, and have not had significant similarity to known genomes.

The second preliminary (i.e., non-optimized) assembly of the *Caenorhabditis* sp. 5 genome derived from the cleaned read data contained 10,120 contigs, with an N50 of 31.4 kb (**Table 3**). It scored equivalently to the first assembly in biological measures of completeness (including mapping to *Caenorhabditis* sp. 5 expressed sequence tags (Elsworth et al., 2011), representation of matches to the proteome of the closely related *Caenorhabditis briggsae* (Stein et al., 2003; Yook et al., 2012), and screening with the Core Eukaryotic Genes Mapping Approach, CEGMA; Parra et al., 2007). Each of these metrics of biological completeness were essentially unaffected by the removal of 25 Mb of contaminating bacterial sequence. The reduction in N50 is partly a product of the removal of the more-easily assembled bacterial data (which had an N50 of ~45 kb). We would expect the N50 to be improved on reassembly under optimal parameters.

**Table 3:** Assembly statistics for *Caenorhabditis* sp. 5.

**Measure**	**Preliminary assembly**	**Contigs removed from preliminary assembly**	**Assembly of data after removal of reads mapping to contaminant contigs**
Span (bp)	160,970,414	25,566,044	135,507,189
Number of contigs^*^	12,264	2,148	10,120
N50 of contigs (bp)	32,806	44,901	31,396
CEGMA completeness	97.58%	–	96.37%
Representation of *Caenorhabditis* sp. 5 EST transcriptome^**^	98.1%	–	98.1%
Representation of *Caenorhabditis* sp. 5 RNA-Seq transcriptome^***^	97.41%	–	97.42%
Matches to *Caenorhabditis briggsae* proteome^****^	79.04%	–	79.04%

* Or scaffolds, as the contigs may contain “N” base calls.

** The Caenorhabditis sp. 5 expressed sequence tag dataset includes 2,265 unigene sequences.

*** The Caenorhabditis sp. 5 RNA-Seq transcriptome assembly contains 30,756 unigene sequences.

**** Caenorhabditis briggsae is the closest fully sequenced Caenorhabditis species to Caenorhabditis sp. 5. Its proteome contains 21,961 entries.

Multi-genome coassemblies can contain errors. One risk with the TAGC plot method is that sequences erroneously constructed or scaffolded may contain DNA from more than one genome. Removal of all of a contig because one part matches an identified undesired contaminant risks discarding good data. We recommend a conservative approach, for example only discarding contigs that are tagged as having their best megablast match to a contaminant if there is no match better than a relatively permissive cutoff to the target taxon. Similarly it is sometimes difficult to tell where the blobs from the contaminants end and that from the target starts. The *Caenorhabditis* sp. 5 example had relatively clear separation between bacterial and nematode blobs, but this should not be expected in every case. Again, a conservative approach is warranted, retaining the maximal amount of target data.

### IDENTIFYING SYMBIONTS AND LATERAL GENE TRANSFERS WITH TAGC PLOTS

As indicated above, TAGC plots are also useful for separating several desired target genomes from a mixed dataset. In the case of bacterial symbionts of eukaryotes, this then permits independent, optimized assembly of host and symbiont. We illustrate this here with data from the sequencing of the genome of the dog heartworm, *D. immitis* (genome size ~95 Mb), which carries an apparently obligate symbiont, the rickettsial alphaproteobacterium *Wolbachia pipientis* wDi (genome size ~1 Mb; Godel et al., 2012). Fragments of the wDi genome are present in the nematode nuclear genome, horizontally transferred from this germline-transmitted symbiont. In this case therefore, simple separation by taxonomic annotation of the contigs may risk confusing true wDi contigs with nuclear insertions. For *D. immitis*, we generated datasets from two different nematodes, including male (“Pavia”; where *Wolbachia* abundance is low) and female (“Athens”; where abundance is higher). In the TAGC plots of the different libraries (**Figure 3**) distinct blobs annotated as Rickettsiales in origin were found at different relative coverage in each library. In the “Athens” library the Rickettsiales wDi blob is clearly separable from the nuclear *D. immitis* blob, as it has approximately 10-fold greater coverage. Also evident in the “Athens” data is a low coverage blob of higher GC content. This blob is derived from the canine host of *D. immitis.* A simple coverage cutoff along with a selection for megablast matches to Rickettsiales resulted in a high-quality wDi read set that generated a much better assembly (reducing the number of contigs from 63 to only two, one of 920 kb and one of 1 kb; Comandatore et al., 2013). Similarly, removal of the dog contamination, and filtering the wDi reads generated a better *D. immitis* assembly. This procedure also usefully left the wDi nuclear insertion-derived read data in the nuclear genome read set, permitting investigation of laterally transferred fragments (Godel et al., 2012).

## DISCUSSION

We have presented an approach to interpreting and cleaning raw high-volume sequence datasets to improve both assembly metrics and biological interpretation. The ideas behind this approach are not new. Difference in GC proportion is used by several raw data quality-control tools, such as fastqc (Andrews, 2013), to identify potential problems in raw read data. Coverage filters are commonly used in genome assembly to remove low- and high-abundance k-mers from de Bruijn graphs to simplify resolution. Taxonomic annotation is commonly used post assembly to identify contaminants. What distinguishes the TAGC plot approach is the combining of these measures in screening preliminary assemblies in the context of targeted sequencing of “contaminated” samples. TAGC plots are very useful in pre-screening pilot datasets before proceeding to bulk sequencing, as they can identify unexpected contamination of target genomes with other DNA. They assist in generating better assemblies by separating different genomes that need different assembly parameter sets into independent assembly projects. Rather than achieve a global optimum that in fact is not at all optimal for each constituent genome, split-data assembly can approach each genome and find local optima. In addition, early removal of contaminant genes from a target assembly can avoid compromising costly downstream analyses with rogue data.

The problem of multi-genome datasets is at the core of the huge effort that has gone in to development of assemblers capable of delivering biologically meaningful results from metagenomic datasets. In a metagenome study, the “target” is usually all the genomes in the environment studied, and an important analytical goal is the identification of which genes in the environment are present on the same replicons, and thus likely to be active within a single membrane-bound organism. To approach the binning of metagenome data, several groups have used approaches similar to TAGC plots, integrating coverage, GC, and taxonomic affinity to propose potential linkages between contigs. Importantly, some authors have in addition used higher-dimensional vectors of base composition patterns than simple nt counts. A major locus of activity has been in the use of multidimensional dinucleotide, trinucleotide and, most commonly, tetranucleotide composition vectors (4NCV; Teeling et al., 2004; Slater and Birney, 2005; Chatterji et al., 2007; Emmersen et al., 2007; Dick et al., 2009; Willner et al., 2009; Ghosh et al., 2011; Lamprea-Burgunder et al., 2011; Brisson et al., 2012; Saeed et al., 2012; Strous et al., 2012). Hexanucleotide counting has also been used to separate simple mixtures of a few species (Hraber and Weller, 2001). Where whole-genome sequence training data are available, 4NCV are extremely powerful in binning new data into “known” groups. Applied *de novo* to metagenomic data, 4NCV can be used to inform hypotheses of association between sequences. The limitation in the 4NCV approach is that the vectors are most informative when derived from long sequences (tens of kilobases) and become less discriminatory when derived from short contigs or reads. The best available 4NCV tool, MetaWatt (Strous et al., 2012), uses machine learning to cluster contigs into bins of coherent coverage, GC proportion, 4NCV, and taxonomic annotation. It has a highly featured graphical user interface that aids exploration and selection of binned data. In our hands, the tool is effective but hard to use with larger eukaryotic datasets, as it over-splits the datasets, and is particularly slow to respond when a large number of bins and their contigs are selected. It is clear that addition of 4NCV (or similar high-dimensional nt pattern information) to the TAGC plot approach could be very valuable, particularly if efficient methods of unsupervised binning could be developed. Other tools designed to split raw or assembled data into bins that putatively derive from distinct species have been proposed that might serve as useful post TAGC-plot approaches. Support vector machines informed by corpora of training data can be used to separate mixed-origin assemblies based prior expectations of species content (Rudd and Tetko, 2005; Emmersen et al., 2007). Another development might be to use a read or k-mer normalization method such as khmer (Brown et al., 2012) to first equalize the effective molarity of the genomes, and then simply use taxonomic matching (and/or 4NCV) to separate the contigs into putative single-genome bins.

The TAGC plot method has been used in several recent genome assembly efforts, largely thus far in nematodes (because of our laboratory’s interests and contacts). We and colleagues have used it in assembly of several species’ genomes, and in isolation of their *Wolbachia* symbionts (Kumar and Blaxter, 2011; Godel et al., 2012; Kumar, 2012; Comandatore et al., 2013; see also http://nematod.es for open access genomes from additional species). Schwarz et al. (2013) used TAGC plots to clean up their *Haemonchus contortus* read sets before assembly. We have also used TAGC plots to examine transcriptome assemblies, though obviously the coverage dimension in these data reflects gene expression levels rather than genome coverage, and have found them useful, particularly when screening infected hosts sequenced to reveal both host and parasite/pathogen transcription (Heitlinger et al., 2013). Edinburgh Genomics^1^ use TAGC plots as a standard part of their data quality-control pipeline, particularly for ecologically or environmentally focused genomics projects where the species of interest is new to genome analysis.

## Conflict of Interest Statement

The authors declare that the research was conducted in the absence of any commercial or financial relationships that could be construed as a potential conflict of interest.

## AUTHOR CONTRIBUTIONS

The TAGC plot software was devised by Mark Blaxter and Sujai Kumar and written by Sujai Kumar in consultation with Mark Blaxter, Martin Jones, Georgios Koutsovoulos, and Michael Clarke. Blobsplorer was written by Martin Jones. The software was tested and improved by Sujai Kumar, Georgios Koutsovoulos, Michael Clarke, and Mark Blaxter. All authors contributed to the writing of the manuscript.

## Footnotes

1 http://genomics.ed.ac.uk
